# TRANSLATIONAL BIOMARKERS FOR AGING AND FRAILTY: IMPORTANT SEX DIFFERENCES

**DOI:** 10.1093/geroni/igae098.1645

**Published:** 2024-12-31

**Authors:** Alice Kane

**Affiliations:** Institute for Systems Biology, Seattle, Washington, United States

## Abstract

Frailty can be measured using a frailty index, which quantifies the accumulation of health-related deficits. Frailty increases the risk of adverse outcomes including hospitalization and mortality, but there are no validated frailty biomarkers and the underlying mechanisms of frailty remain unknown. Additionally, women are more frail than men at all ages, despite having a lower risk of mortality, but sex differences in frailty are poorly understood. We have assessed health outcomes, including frailty, in male and female C57BL/6 mice from middle age to natural death. We collected blood and stool samples at each timepoint, for untargeted plasma metabolomics, blood DNA methylation assessment and gut microbiome analysis. As in humans, female mice have higher frailty scores than males in middle-to-late life. Sex explains more variability than age or frailty in our DNA methylation data, and preliminary analysis identifies 100s of sex-specific differentially methylated regions with frailty. Metabolomics analysis identifies a set of 104 metabolites (enriched for amino acid and lipid pathways) associated with frailty, independent of sex and age. Additionally, we identify many sex-specific metabolomic markers for frailty, including creatine for females and FAD for males. Analysis of the microbiome data identifies microbes that are both positively and negatively associated with frailty across sexes including A. colihominis. Integrated analysis across data-types is ongoing. Overall, we see clear sex differences in the association of frailty with molecular outcomes in mice. Further investigation of these factors, and validation in human cohorts, will identify determinants and biomarkers of frailty in both sexes.

